# Kalkaneoplastie mit Radiofrequenzzementierung nach Ballonaufrichtung

**DOI:** 10.1007/s00113-023-01365-2

**Published:** 2023-10-24

**Authors:** J. Rathjen, M. Völlmecke, D. Bieler, A. Franke, E. Kollig

**Affiliations:** Klinik für Unfallchirurgie und Orthopädie, Hand- und Wiederherstellungschirurgie, Verbrennungsmedizin, Bundeswehrzentralkrankenhaus Koblenz, Rübenacherstraße 170, 56072 Koblenz, Deutschland

**Keywords:** Zementaugmentierte Osteosynthese, Fußchirurgie, Fersenbein, Calcaneusfraktur, Minimalinvasive Chirurgie, Cement augmentated osteosynthesis, Foot surgery, Heel bone, Calcanela fracture, Minimally invasive surgery

## Abstract

Als operatives Standardverfahren von komplexen Kalkaneusfrakturen gilt die offene Reposition und interne winkelstabile Plattenosteosynthese über einen lateralen Zugang. In jüngerer Zeit wurden auch Optionen für minimalinvasive und perkutane Verfahrensstrategien vorgestellt [7, 4]. Als mögliche Verfahrensalternative für eine gedeckte, operative Versorgung von Fersenbeinbrüchen wird in diesem Zusammenhang die Kalkaneoplastie diskutiert und angewendet [5]. In der hier vorgestellten Fallserie von 5 versorgten, komplexen Kalkaneusfrakturen wurde ein Ballonkatheter zur perkutanen Reposition verwendet, um das Alignment des Kalkaneus wiederherzustellen.

Anschließend erfolgten das Einbringen von PMMA-Zement in Radiofrequenzanwendung und die Osteosynthese mittels perkutanen, kanülierten Schrauben. Diese Technik erlaubt nach Vicenti eine stabile Reposition und Retention mit früher Teilbelastung bei insgesamt geringer Komplikationsrate [17].

## Abstract (en)

The standard surgical procedure for complex calcaneal fractures is open reduction, internal reduction and internal stable angle plate osteosynthesis via a lateral approach. More recently, options for minimally invasive and percutaneous surgical strategies have been presented [4, 7]. As a possible procedural alternative for a covered, surgical treatment of calcaneal fractures, calcaneoplasty is discussed and applied in this context [5]. In this case series of five complex calcaneal fractures presented here, a balloon catheter was used for percutaneous reduction to restore the alignment of the calcaneus.

This was followed by placement of PMMA cement in radiofrequency application and osteosynthesis using percutaneous cannulated screws. This Vicenti technique allows stable reduction and retention with early partial weight bearing with an overall low complication rate [17].

## Hintergrund

Als operatives Standardverfahren von komplexen Kalkaneusfrakturen gilt die offene Reposition und interne winkelstabile Plattenosteosynthese über einen lateralen Zugang. Bereits ab 1900 wurden minimalinvasive Verfahren zur Versorgung von Böhler, Wolff und v. a. Westhues beschrieben, dessen Technik bei „Tongue-type“-Frakturen als Standardverfahren zur minimalinvasiven Versorgung gilt [2, 6, 18]. In jüngerer Zeit wurden weitere Optionen für minimalinvasive und perkutane Verfahrensstrategien vorgestellt [4, 7]. Als mögliche Verfahrensalternative für eine gedeckte, operative Versorgung von Stressfrakturen wird auch die Kalkaneoplastie diskutiert und angewendet [5]. In der hier vorgestellten Fallserie von 5 operativ versorgten, komplexen Kalkaneusfrakturen wurde ein Ballonkatheter zur perkutanen Reposition verwendet, um das Alignment des Kalkaneus wiederherzustellen. Anschließend erfolgten das Einbringen von PMMA(Polymethylmethacrylat)-Zement in Radiofrequenzanwendung und die Osteosynthese mittels perkutanen, kanülierten Schrauben.

Diese Technik erlaubt nach Vicenti eine stabile Reposition und Retention mit früher Teilbelastung bei insgesamt geringer Komplikationsrate [17].

## Patienten und Methoden

Im Zeitraum von Dezember 2019 bis September 2020 wurden 4 Patienten (Tab. 1) mit insgesamt 5 Kalkaneusfrakturen mittels perkutaner Radiofrequenzballonkalkaneoplastie operativ versorgt. Drei Patienten waren männlich, eine weiblich. Ein Mann hatte beidseitige Kalkaneusfrakturen.

**Table 1:** 

	Frakturursache	Frakturtyp	Klassifikation nach Sanders	Komorbiditäten
Patient 1	Leitersturz aus 2,5 m Höhe	Links: komplexer Joint-Depression-Typ	IIIAC	Nikotinabusus, pAVK Stadium IV, thrombotischer Verschlusses des vorbestehenden femorokruralen Bypass rechts, vor definitiver Frakturversorgung revaskularisiert
		Rechts: Joint-Depression-Typ	IIB	
Patient 2	Balkonsturz aus 2m Höhe	Joint-depression-Typ mit erheblicher Weichgewebsschwellung und 2 × 2cm trockener Nekrose medial	IIB	Asthma bronchiale, arterielle Hypertonie, Adipositas Grad II, Nikotinabusus
Patient 3	Treppensturz mit axialem Anprall	Mehrfragmentäre, intraartikuläre Fraktur vom Joint-Depression-Typ mit kombinierter Tongue-Type-Komponente	IIIBC	–
Patientin 4	Treppensturz mit axialem Anprall	Mehrfragmentäre, dislozierte Kalkaneusfraktur vom Joint-Depression-Typ mit großem Fragment aus der hinteren Kammer des USG, tief in das Corpus calcanei imprimiert mit großem Fragment aus der hinteren Kammer des USG, tief in das Corpus calcanei imprimiert	IIC	Osteoporose unter Alendronsäuretherapie

*pAVK* periphere arterielle Verschlusskrankheit

Das mittlere Alter betrug 50 Jahre (33 bis 66 Jahre). Alle Patienten wurden stationär aufgenommen. Aufgrund der frakturbedingten Weichteilsituation wurden bei 2 Patienten zunächst eine geschlossene Reposition und die Anlage eines Fixateur externe durchgeführt. Dieser wurde gelenküberbrückend angelegt, insbesondere um die Länge des Kalkaneus wiederherzustellen. Bei den anderen Frakturen konnte auf die Anlage eines temporären Fixateur externe bei fehlender grober Dislokation verzichtet werden. Hier erfolgten zunächst konservative abschwellende Maßnahmen.

Alle Frakturen waren Folgen von Stürzen. Dabei handelte es sich um 2 Treppenstürze im häuslichen Umfeld, einen Sturz aus ca. 2 m Höhe von einem Balkon sowie um einen Leitersturz aus ca. 2,5 m Höhe.

An Vorerkrankungen bestanden im Patientenkollektiv eine Osteoporose unter oraler Osteoporosetherapie mit Alendronsäure, Asthma bronchiale, arterielle Hypertonie, Adipositas Grad II, Nikotinabusus, eine periphere arterielle Verschlusskrankheit vom Becken-Bein-Typ Stadium IV nach Fontaine, die vor der osteosynthetischen Versorgung des Kalkaneus rechtsseitig eines femorokruralen autologen V.-saphena-magna-Bypass bedurfte.

**Figure 1:**
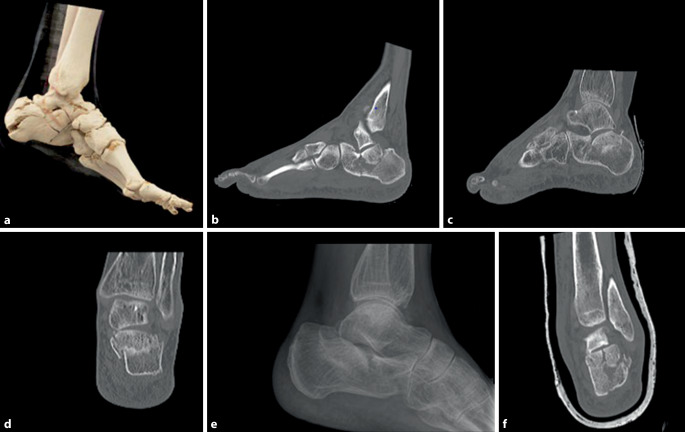

In der konventionellen Bildgebung („mortise view“, Fuß lateral und Broden-Aufnahme) und Schnittbilddiagnostik wurden Kalkaneusfrakturen, teilweise auch komplexe mehrfragmentäre, intraartikuläre Frakturen vom Joint-Depression-Typ nach Essex-Lopresti identifiziert. In einem weiteren Fall bestand ein Gelenkfragment aus der hinteren Kammer des unteren Sprunggelenkes, das tief in das Corpus calcanei imprimiert war (Abb. 1).

In der Zusammenschau aller vorliegenden Befunde wurde das therapeutische Vorgehen in einer unfallchirurgisch-radiologisch interdisziplinären Fallkonferenz mit Röntgenbilddemonstration im Kollegium und anschließend mit den Patienten erörtert. Aufgrund der vorliegenden Weichteilsituationen sowie der vorhandenen Nebenerkrankungen wurde sich in diesen Fällen für die Durchführung einer minimalinvasiven Methode mittels perkutaner radiofrequenzzementaugmentierter Ballonkalkaneoplastie, vergleichbar mit dem Verfahren der Kyphoplastie, entschieden.

## Operatives Vorgehen: radiofrequenzzementaugmentierte Ballonkalkaneoplastie

**Figure 2:**
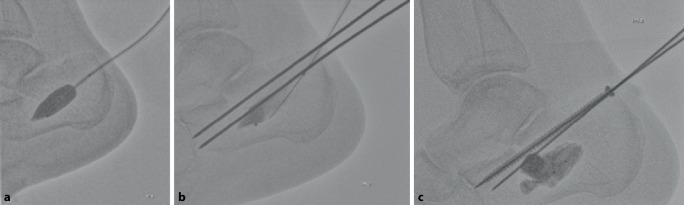

**Figure 3:**
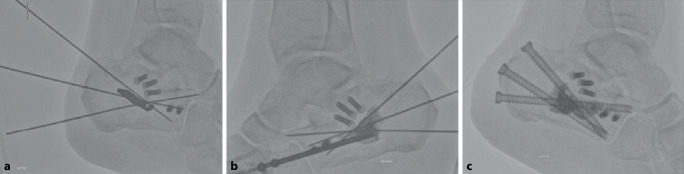

**Figure 4:**
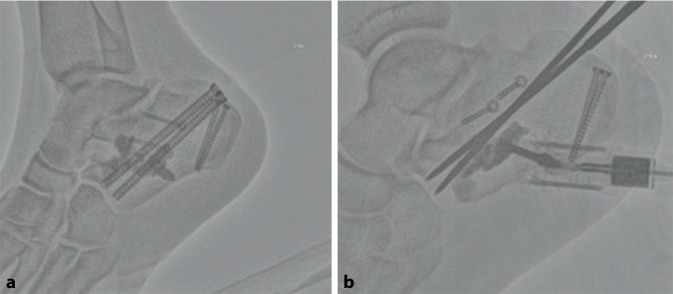

Auf das Aktivieren einer Blutsperre kann bei dem perkutanen Vorgehen in der Regel verzichtet werden. Bei der hier vorgestellten Serie erfolgte die operative Versorgung in Zusammenarbeit mit der Radiologie unter Nutzung eines Durchleuchtungsroboters für minimalinvasive Prozeduren (ARTIS pheno®, Fa. Siemens Healthcare GmbH, Erlangen, Deutschland) in kontralateraler Seitenlage nach präoperativer „Single-shot“-Antibiose mit einem Zweitgenerationscephalosporin. Zur Wiederherstellung des Alignments hinsichtlich Länge und Achse hat sich eine in die Pars posterior calcanei von dorsal eingebrachte Schanz-Schraube mit einem T‑Griff als Manipulations- und Repositionshilfe sehr bewährt. Die Feinreposition gelingt mittels über Stichinzisionen eingebrachte K‑Drähte (mindestens Stärke 2,0 mm), die auch zur temporären Transfixation z. B. gegen den Talus genutzt werden. Ein typisch längsgespaltener, hinterer subtalarer Gelenkblock kann nach Desimpaktieren der Fragmente bereits jetzt mit exakter Wiederherstellung der Gelenkfläche mit queren Schrauben gestellt werden. Der Ballonkatheter wird über einen Führungsdraht via Stichinzision von lateral in den subtalaren Defektbereich eingebracht und exakt unter dem Gissane-Winkel positioniert und sukzessive mit bis zu 6 bar mit kontrastierter (konventionelles i.v.-Kontrastmittel) NaCl-Lösung gefüllt (Abb. 2). Dabei wird der Ballon unter dem imprimierten Fragment positioniert und hebt – ähnlich einer Kyphoplastie – durch die sukzessive Füllung unter BV-Kontrolle das gelenktragende Hauptfragment an. Die Ballonaufrichtung erfolgte bei jeder der geschilderten Versorgungen unter manometrischer Kontrolle, wobei ein abruptes Vorgehen zu vermeiden ist, da es sonst zum Platzen des Ballons an Fragmentkanten kommen kann. Anschließend wurde zusätzlich über kleine Stichinzisionen von lateral vorwiegend über dem Sinus tarsi unter Durchleuchtungskontrolle mit dem Angioroboter mit einem kleinen Elevatorium eine exakte Feinreposition vorgenommen. Mithilfe der 3D-Navigation wird jeweils eine genaue dreidimensionale Darstellung der Reposition bestätigt, um eine exakte anatomische Stellung zu gewährleisten. Bei zufriedenstellender Reposition werden die Hauptfragmente mit Kirschner-Drähten temporär transfixiert (Abb. 3). Zwei weitere parallele Drähte werden dabei vom Tuber calcanei eingebracht und bis zum Processus anterior vorgebohrt. Anschließend wird der Ballon entfernt und der entstandene Substanzdefekt mit Zement (StabiliT^©^ Bone Cement, Fa. Merit Medical, South Jordan, UT, USA) in Radiofrequenztechnik aufgefüllt. Diese erlaubt die Einstellung der Viskosität und damit eine exaktere Zementierung. Über die parallelen Führungsdrähte werden kanülierte Schrauben (Asnis® III, Fa. Stryker, Freiburg, Deutschland) über die Frakturzone in den Processus anterior durch den noch weichen Zement im Sinne einer Verbundosteosynthese (Abb. 4) eingebracht. Anschließend werden vor Aushärten des Zementes alle Kirschner-Drähte entfernt.

**Figure 5:**
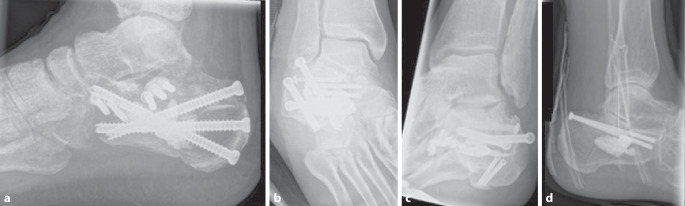

**Figure 6:**
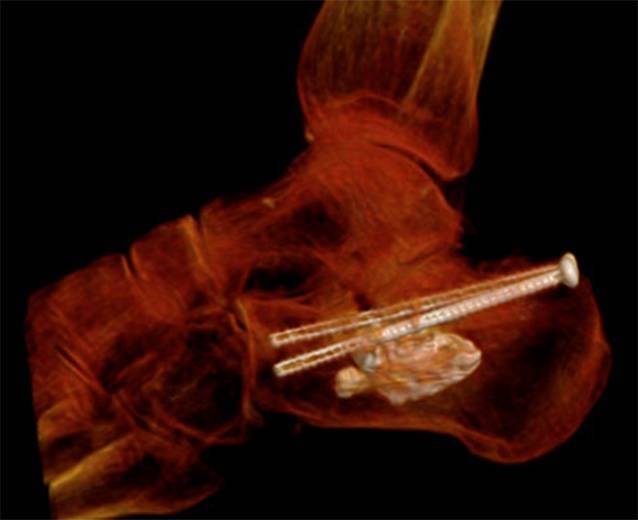

Auch bei komplexeren und/oder kombinierten Frakturmorphologien kann diese Technik angewandt werden. Hierbei empfiehlt es sich, z. B. zunächst die Tongue-Type-Komponente der Pars posterior ebenfalls perkutan über kleine Stichinzisionen zu reponieren und präliminar mit Bohrdrähten oder einer Repositionszange zu halten. Es schließt sich das Einjustieren des ggf. vorher wiederhergestellten Gelenkblocks gegen die korrespondierende Gelenkfläche des Talus mittels Ballonkatheter an. Die gesamte Versorgung erfolgt minimalinvasiv über kleine Stichinzisionen von lateral. Eventuell aus dem Defekt ausgetretener Zement sollte umgehend entfernt werden, um mechanischen Irritationen hierdurch vorzubeugen. Grundsätzlich könnte der eingebrachte Zement den Frakturlinien folgend nach medial, plantar, lateral und in das Subtalargelenk austreten; dies ist in der Fallserie nicht vorgekommen. Ein entsprechend vorsichtiges Einbringen des Zements unter Viskositätskontrolle via Radiofrequenztechnik und Durchleuchtung kann unerwünschte Extravasate verhindern. Bei der abschließenden Bildgebung des operativen Ergebnisses ist auf die korrekte Einstellung des Gissane-Winkels in der seitlichen Durchleuchtung sowie eine stufenfreie Abbildung in der Broden-Aufnahme zu achten (Abb. 5). Abb. 6 zeigt beispielhaft einen abschließenden Scan mit dem Durchleuchtungsroboter (Abb. 6). Die Weiterbehandlung erfolgt für 8 Wochen in einer rückfußentlastenden Orthese (z. B. nach Settner) unter medikamentöser Thromboembolieprophylaxe mit regelmäßigen Blutbildkontrollen (z. A. HIT-II-Reaktion). Vier und 8 Wochen postoperativ werden konventionelle Röntgenkontrollen angefertigt (seitlich, axial und Broden-Projektion), im Bedarfsfall eine CT.

## Ergebnisse

In allen Fällen wurden intraartikuläre Kalkaneusfrakturen vom Joint-Depression-Typ nach Essex-Lopresti identifiziert. In einem Fall lag begleitend ein medialseitiger erheblicher Weichgewebsschaden vor; dieser entwickelte sich im Verlauf zu einer trockenen Nekrose.

Im stationären Verlauf kam es zu keinen prä-, intra- oder postoperativen Komplikationen. Ein Patient bedurfte bei vorbestehender pAVK und Bypass-Verschluss zunächst einer femurokuralen Revaskularisierung, sodass sich der Eingriff am Fersenbein um 7 Tage verzögerte. Unmittelbar postoperativ konnten alle Patienten unter physiotherapeutischer Anleitung und Thromboembolieprophylaxe mit niedermolekularem Heparin zunächst an Unterarmgehhilfen mit Entlastung des Fersenbeins in Rückfußentlastungsorthesen auf Stations- und Krankenhausebenen mobilisiert werden. In keinem Fall trat eine Wundinfektion auf. In allen Fällen gelang es, unter suffizienter Analgesie die Mobilisation zu erreichen.

Für die klinische und radiologische Verlaufskontrolle im postoperativen Verlauf konnten leider nur 2 Patienten mit insgesamt 3 Kalkaneusfrakturen aus dem BGlichen Heilverfahren wieder einbestellt werden.

**Figure 7:**
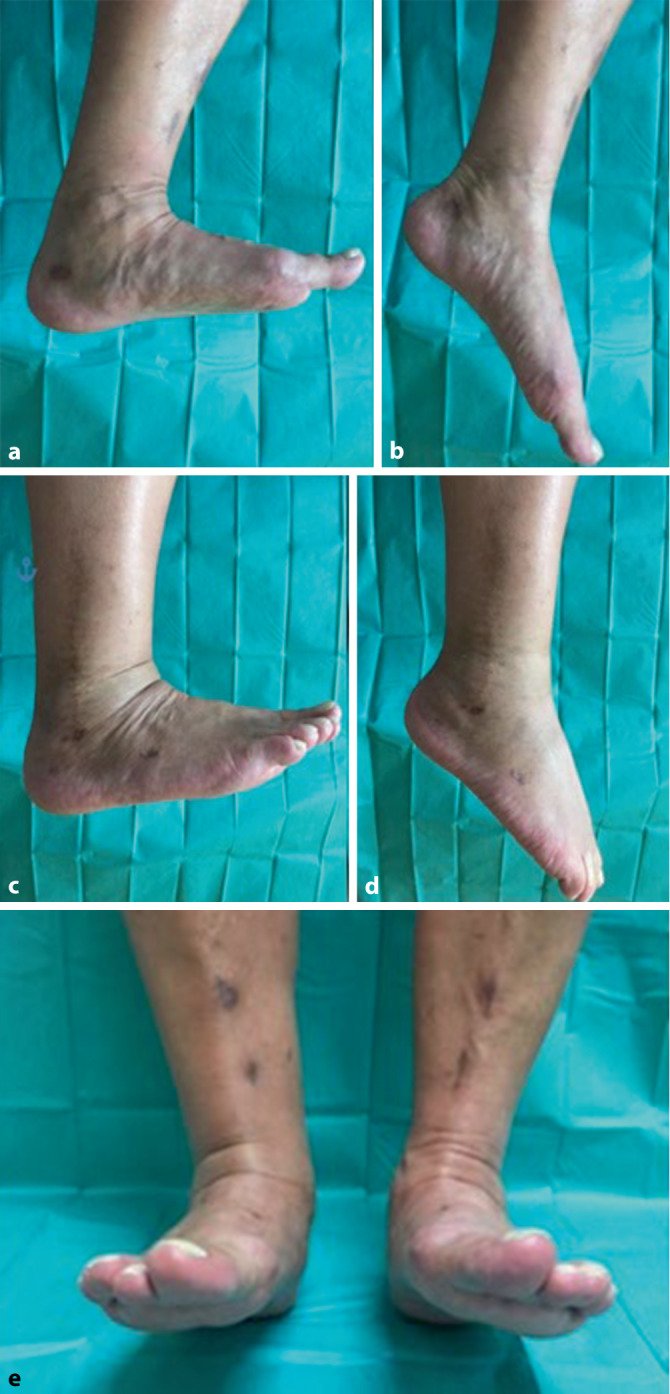

Hierbei zeigten sich bei beiden reizlose Narbenverhältnisse. Es wurde zunächst die Entlastung beider Fersenbeine in den rückfußentlastenden Orthesen an Unterarmgehhilfen unter medikamentöser Thromboembolieprophylaxe durchgeführt. Begleitend erfolgte eine physiotherapeutische Übungsbehandlung. Bei beiden Patienten war nach 8 Wochen und regelrechten Durchbauungszeichen im konventionellen Röntgenbild die zügige, programmierte Aufbelastung bis zur Vollbelastung möglich. Letztlich war die axiale Vollbelastung in vorkonfektioniertem Schuhwerk mit orthopädischen Einlagen mit Fersenweichbettung nach Pedobarographie möglich. Die Verlaufskontrolle 4 Monate nach Operation und im mehrjährigen Intervall zeigte für beide Patienten ein zufriedenstellendes funktionelles Ergebnis im oberen und insbesondere auch im unteren Sprunggelenk für die Pronation und Supination (Abb. 7). Patientin Nr. 4 berichtete 3 Jahre postoperativ über die Möglichkeit, 6 km täglich spazieren gehen zu können. Dabei trage sie vorkonfektionierte Turnschuhe mit einer fersenweichbettenden Einlage.

Patient 1 kann ebenfalls in vorkonfektioniertem Schuhwerk beide Fersen axial voll belasten. Lediglich das Klettern auf Leitersprossen mit den Vorfüßen bereite ihm Schmerzen. Dabei sei der linke Fuß schmerzhafter als der rechte. Dazu passend stellt sich im konventionellen Röntgenbild im lateralen Strahlengang eine Arthrose insbesondere des unteren Sprunggelenks dar.

**Figure 8:**
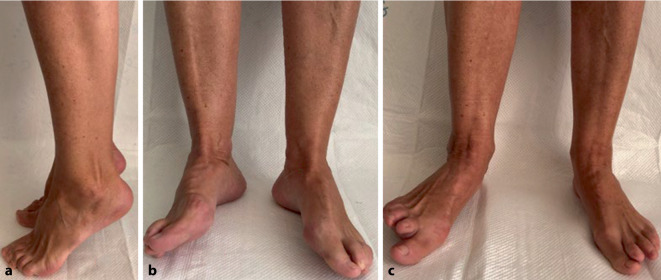

**Figure 9:**
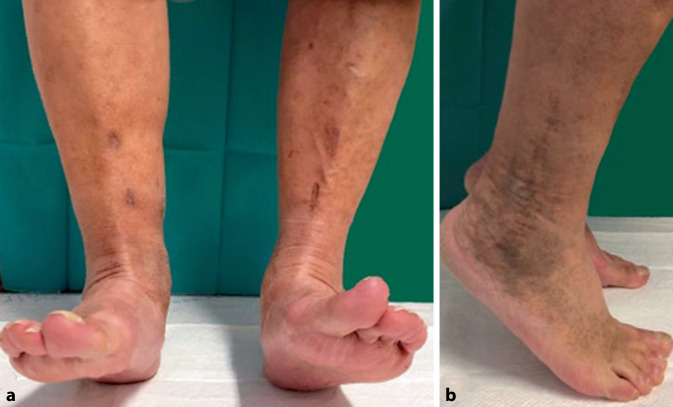

**Figure 10:**
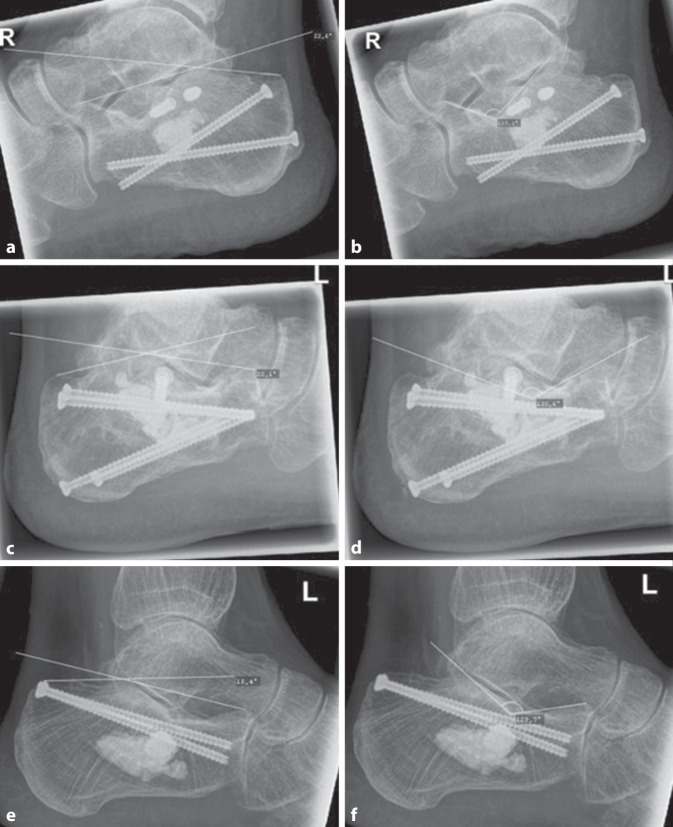

Die Abb. 8 stellt das funktionelle klinische Ergebnis von Patientin 4 im mehrjährigen Verlauf dar:

Die Abb. 9 stellt das funktionelle klinische Ergebnis von Patient 1 im mehrjährigen Verlauf dar:

Die Abb. 10 stellt die gemessenen Böhler- und Gissane-Winkel von Patient 1 und Patientin 4 im mehrjährigen Verlauf dar.

## Diskussion

Die Kalkaneusfraktur ist selten, stellt jedoch mit einem 60- bis 75 %igen Anteil an tarsalen Frakturen den häufigsten Bruch der Fußwurzel dar. Insgesamt sind Männer bis zu 5‑mal häufiger betroffen als Frauen und verletzen sich im Alter zwischen 30 und 50 Jahren zu 90 % im erwerbsfähigen Berufsleben [11, 20]. Ebenso stellt es sich in dieser Falldemonstration dar.

Komplexe Kalkaneusfrakturen hinterlassen ohne adäquate Behandlung in relevantem Umfang posttraumatische Funktionseinschränkungen, erhebliche Gangbildstörungen und sekundäre Arthrosen, vorwiegend im unteren Sprunggelenk. Die berufliche Reintegration von Unfallverletzten gestaltet sich regelmäßig schwierig, es verbleibt regelmäßig eine relevante Minderung der Erwerbsfähigkeit mit entsprechenden Leistungsansprüchen (Auerswald et al., 2014). Dadurch ergibt sich die volkswirtschaftliche Bedeutung dieser Verletzung. Im Fall einer berufsgenossenschaftlich versicherten Verletzung ist von einer durchschnittlichen Dauer des Heilverfahrens von 8 bis 10 Monaten auszugehen. In 13 % aller Fälle tritt eine dauerhafte Arbeitsunfähigkeit ein. Die durchschnittliche Minderung der Erwerbsfähigkeit (MdE) liegt bei 19 %. Daraus ergeben sich volkswirtschaftlich relevante Kosten in Höhe von 25.540 €/Fall bis zum ersten Rentengutachten [11].

Das Heilverfahren im hier dargestellten Fall dauerte insgesamt 7 Monate und ist damit kürzer als der genannte Durchschnitt. Arbeitsfähigkeit bestand bereits nach 5 Monaten.

Therapeutisch kommen grundsätzlich eine konservative Behandlung und eine operative Versorgung in Betracht. Ausgenommen die isolierte Sustentaculum-Fraktur, kann bei nichtdislozierten Brüchen oder bei Kontraindikationen zur operativen Versorgung die konservative Therapie mit Entlastung für 6 bis 12 Wochen an Unterarmgehstützen erfolgen. Dabei muss berücksichtigt werden, dass es durch Verzicht auf eine anatomische Rekonstruktion der Hauptgelenke im Rückfuß zu Gelenkinkongruenz mit wahrscheinlicher posttraumatischer Arthrose und u. U. zu Fehlheilungen in Form von Rückfußvarus oder seltener -valgus, Plattfuß oder Rückfußverbreiterungen kommen kann [14].

Die offene Reposition und interne Fixierung mittels Schraubenosteosynthese des Kalkaneus, inklusive Sustentaculum-Schrauben oder winkelstabiler Plattenosteosynthese über den erweiterten lateralen Zugang, stellt das bewährte Standardverfahren zur operativen Versorgung dislozierter, intraartikulärer Frakturen dar und bietet den Vorteil der guten anatomischen Übersicht ins untere Sprunggelenk sowie guter Repositionsergebnisse. Ziel der operativen Versorgung dislozierter Kalkaneusfrakturen ist die exakte anatomische Wiederherstellung der korrekten Höhen- und Achsenverhältnisse des Rückfußes sowie der Gelenkkongruenz [22]. Klinische und biomechanische Arbeiten konnten nachweisen, dass das Fehlen der Sustentaculum-Schraube zu einer schlechteren Biomechanik führt, die zum Einbrechen des Böhler-Winkels führen kann [10, 12]. Erwähnenswerte Komplikationen sind frühe Wundheilungsstörungen und Wundinfektionen, Implantatversagen und im Verlauf posttraumatische Arthrosen mit notwendigen Folgeoperationen bis hin zur Arthrodese des unteren Sprunggelenks. Die häufigste Frühkomplikation ist eine Wundinfektion, die jedoch in den meisten Fällen mittels konservativer Therapie kontrolliert werden kann [3]. Das häufige Auftreten von frühen Weichteilinfekten lässt sich durch die geringe Weichteildeckung und die spezielle Gefäßversorgung im Gebiet des lateralen Zugangs erklären.

Bei einfachen Frakturformen kann auch eine arthroskopisch und/oder bildwandlergestützte, minimalinvasive, perkutane Versorgung durchgeführt werden [4, 9]. Über ein anterolaterales und posterolaterales Portal können die Retention und Fixierung nach geschlossener Reposition mithilfe des Westhues-Repositionsmanövers erfolgen. Unter arthroskopischer Sicht können Feinkorrekturen mit perkutan eingebrachten Stößeln oder Kirschner-Drähten unter zusätzlicher Bildwandlerkontrolle erreicht werden und die Retention der Fragmente beispielsweise mit Kortikalisschrauben über Stichinzision durchgeführt werden [21]. Der minimalinvasive Zugang hat den Vorteil der geringeren Weichteilkompromittierung und niedrigerer Komplikationsrate.

Als weitere Alternative zur geschlossenen Reposition wird seit Kurzem die Ballonkalkaneoplastie bei Frakturformen mit großen solitären Fragmenten angegeben. Das Verfahren ähnelt in seinen Grundzügen der Kyphoplastie. Entsprechend hoch ist die hierauf fußende Expertise im Einsatz des Durchleuchtungsroboters. Diese Technik erlaubt eine stabile Reposition und Retention mit früher Teilbelastung bei gutem klinischem Ergebnis mit insgesamt geringer Komplikationsrate [17]. In einer Multizenterstudie haben Vicenti et al. die Ballonkalkaneoplastie mit dem Standardverfahren der offenen Reposition und Osteosynthese sowie der geschlossenen Reposition mittels Fixateur externe bei intraartikulären Kalkaneusfrakturen verglichen. Die Ergebnisse zeigten zwar eine Tendenz, dass die Ballonkalkaneoplastie, verglichen mit einer offenen Reposition und osteosynthetischer Versorgung, zu besserem funktionellem Outcome führt, der Unterschied jedoch nicht statistisch signifikant war. Zudem war die Fallzahl von 16 durchgeführten Kalkaneoplastien bei insgesamt 202 Fällen gering [16]. Die Fallzahlen in der Literatur zur Ballonkalkaneoplastie sind bisher überschaubar. Für eine Indikationsstellung sollten die Weichteilverhältnisse und weitere Risikofaktoren des Patienten berücksichtigt werden, die ein minimalinvasives Verfahren günstiger erscheinen lassen. Frakturmorphologisch sollte zur Versorgung mittels Ballonkalkaneoplastie das imprimierte Frakturfragment groß genug sein und keine allzu große Trümmerzone aufweisen, um eine suffiziente Aufrichtung mittels Ballon zu erreichen. Es steht außer Frage und wurde vielfach in wissenschaftlichen Arbeiten nachgewiesen, dass eine möglichst anatomische Reposition die Grundlage für ein gutes Outcome ist. Insbesondere muss der Gelenkbereich anatomisch optimal reponiert werden.

Vicenti empfiehlt die Ballonreposition bei Frakturen mit großem Gelenkfragment und geringer Fragmentierung [17]. Dies ergibt sich auch aus einer systembedingten Überlegung: Ein Ballon, der aufgeblasen wird, dehnt sich ungerichtet aus und braucht damit einen Gegenhalt, um zielgerichtet zu funktionieren. Abgesehen von häufigen Frakturen des Fersenbeinbodens findet sich bei Depression-Typ-Frakturen fast immer ein laterales ausgebrochenes Gelenkfragment. Dieses offensichtliche Problem wird auch in der Arbeit von Jacquot angesprochen [8]. Er versuchte, die laterale Wand während des Aufblasens mit einer Klammer von außen zu stabilisieren. Eine Verbreiterung und Verkippung des lateralen Gelenkfragmentes bleibt jedoch problematisch.

In der Literatur finden sich keine prospektiven randomisierten vergleichenden Studien, die einen Vorteil einer Zementauffüllung bezüglich sekundärer Dislokation der Gelenkfragmente nachweisen. Das Fersenbein zeigt aufgrund seines hohen Spongiosaanteils grundsätzlich eine gute knöcherne Heilung, vorausgesetzt wir erreichen eine gute Reposition und eine stabile Osteosynthese [13]. Die Technik ähnelt in Grundzügen einer Spongiosaplastik bzw. kann mit einer Augmentation mit trikortikalen Spänen verglichen werden, um den „Defekt“ unterhalb der Gelenkfragmente auszufüllen. Hier finden sich in der Literatur jedoch widersprüchliche Ergebnisse in Metaanalysen, wobei die Aussagekraft der Ergebnisse aufgrund geringer Fallzahl und Datenlage nicht aussagekräftig ist [15, 19]. Grundsätzlich ist festzuhalten, dass es bei typischen Fersenbeinfrakturen zu einer mehr oder weniger starken Kompression und Impaktion im spongiösen Bereich kommt. Computertomographisch finden sich Defektzonen und v. a. nach Reposition des (Tongue‑/Depression‑)Gelenkfragments deutliche Höhlenbildungen im zentralen Korpusbereich nach Anheben der impaktierten Fragmente. Häufig finden sich ein Ausbruch der lateralen Wand und eine Varusfehlstellung. Hier sehen wir den Vorteil der Ballonkalkaneoplastie mit Zementaugmentation der knöchernen Defekte.

In jüngster Vergangenheit wurden in unserer Klinik 4 Patienten mit insgesamt 5 Kalkaneusfrakturen mittels Ballonkalkaneoplastie versorgt. Bereits intraoperativ erlaubt eine dreidimensionale Darstellung der Frakturreposition mittels 3D-Navigation oder mit dem Angioroboter eine genaue anatomische Wiederherstellung der Gelenkflächen. Die ersten Ergebnisse im Hinblick auf das funktionelle Outcome und die Komplikationsraten im kurzzeitigen Verlauf sind vielversprechend. Insbesondere bei vorliegender Weichteilproblematik sowie bei bestehenden Nebenerkrankungen, die bei offen chirurgischem Vorgehen eine erhöhte Komplikationsrate erwarten lassen, ist die perkutane Radiofrequenzballonkalkaneoplastie eine Alternative zum bewährten offen-chirurgischen Standardverfahren. In der eigenen Kohorte traten keine postoperativen Komplikationen auf. Dieser Artikel stellt eine erste kurze Falldarstellung des neuen Operationsverfahrens aus der eigenen Klinik dar. Für weiterführende Rückschlüsse mit wissenschaftlicher Aussagekraft ist eine höhere Fallzahl mit einem belastbaren Follow-up auf der Zeitachse nötig. Dabei sollte im einjährigen, besser 2‑jährigen Verlauf eine klinisch-radiologische Kontrolle mit Ausmessen der Gissane- und Böhler-Winkel im lateralen konventionellen Röntgenbild des operierten Kalkaneus erfolgen. Die Verlaufskontrolle des Operationserfolges ist vor dem Hintergrund notwendig, dass ein Einbrechen des Böhler-Winkels nachgewiesenermaßen zu einer schlechteren Biomechanik führt [10, 12]. Eine standardisierte computertomographische Darstellung aus reinem akademischem Interesse zur Statuserhebung arthrotischer Gelenkveränderungen zu Studienzwecken ist dafür unserer Ansicht nach aus strahlenhygienischen Gründen nicht vertretbar. Bei persistierenden klinischen Beschwerden kann eine ergänzende Computertomographie zur genauen Darstellung der Gelenkflächen und arthrotischer Veränderungen durchgeführt werden.

Die Kalkaneoplastie mit Radiofrequenzzementierung nach Ballonaufrichtung stellt eine Alternative für minimalinvasive und perkutane Verfahrensstrategien zum operativen Standardverfahren von komplexen Kalkaneusfrakturen mittels Plattenosteosynthese über einen lateralen Zugang dar.

## Fazit für die Praxis

- Die Kalkaneusfraktur ist insgesamt eine seltene Verletzungsentität, jedoch die häufigste Fraktur der Fußwurzel.
- Die Kalkaneusfraktur hat eine hohe volkswirtschaftliche Relevanz mit häufig verbleibenden posttraumatischen Funktionseinschränkungen und Minderung der Erwerbsfähigkeit.
- Neben der offenen Reposition und internen Fixierung mittels Plattenosteosynthese über den lateralen Standardzugang bietet die Ballonkalkaneoplastie mit Radiofrequenzzementierung eine moderne Alternative zur Versorgung mit geringen Komplikationsraten insbesondere bei kritischer Weichgewebssituation.
